# Prey-Base Does Not Influence Breeding Success in Eagle Owls (*Bubo bubo*) in Judea, Israel

**DOI:** 10.3390/ani12101280

**Published:** 2022-05-17

**Authors:** Ezra Hadad, Motti Charter, Jakub Z. Kosicki, Reuven Yosef

**Affiliations:** 1 Israel Nature and Parks Authority, 3 Am Ve’Olamo St., Jerusalem 95463, Israel; ezra.hadad9@gmail.com; 2 Department of Geography and Environmental Studies, University of Haifa, Mount Carmel, Haifa 3498838, Israel; mcharter@geo.haifa.ac.il; 3 Shamir Research Institute, Katzrin 1290000, Israel; 4 Department of Avian Biology and Ecology, Adam Mickiewicz University, Uniwersytetu Poznańskiego 6, 61-614 Poznań, Poland; kubako@amu.edu.pl; 5 Ben Gurion University of the Negev, Eilat Campus, Eilat 8810201, Israel; 6 Department of Environmental Sciences, Savitribai Phule Pune University, Pune 411007, India

**Keywords:** diet, mammal, avian, gradient, latitude, bats

## Abstract

**Simple Summary:**

We studied the diet and breeding success of Eagle Owls (*Bubo bubo*) at 14 nests in the Judea region, Israel. A total of 9461 prey items were identified and although mammals and birds dominated, there were also reptiles, amphibians, and invertebrates. The gradient of diversity of mammalian prey decreased from west to east; and avian prey increased correspondingly. The index of prey species diversity had no relationship with breeding success. The prey-base of the Eagle Owls helped identify the changes in geographic distributions of several species. The Eagle Owl’s diet emphasizes its generalist foraging habits, but pairs may be species-specific specialists. This adaptation is especially important in a fast-developing and congested country like Israel and probably allows the species to subsist in the region.

**Abstract:**

The diet and breeding success of Eagle Owls (*Bubo bubo*) have been suggested to vary at different latitudes. However, it is still unclear whether and how these relationships exist at lower latitudes outside of Europe. We therefore studied the diet and breeding success of Eagle Owls during four breeding seasons at 14 nests in the Judea region, Israel. Of a total of 9461 prey items were identified; mammals (N = 6896, 35 species; 72.89%, biomass 62.3%) and birds (N = 2255, 55 species, 23.83%; biomass 36.0%) predominated the prey-base. We found that the gradient of diversity of the mammalian prey decreased from west to east; and avian prey increased from east to west. The index of species diversity, H’ for all prey, had no relationship with breeding success. The prey-base of the Eagle Owls helped identify the changes in geographic distributions of several species. Marbled polecat (*Vormela peregusna*), especially threatened, appear to be relatively abundant, as are brown rats (*Ratttus norvegicus*) which were previously considered to be restricted to the coastal regions. In addition to Egyptian fruit bats (*Rousettus aegyptiacus*), the Eagle Owls also preyed on nine different species of insectivorous bats, several of which appear to have enlarged their geographic distribution within Israel. The Eagle Owl’s diet emphasizes its generalist foraging habits, but pairs may be species-specific specialists. This adaptation is especially important in a fast-developing and congested country like Israel, because a generalist hunting strategy probably allows the species to subsist in the region.

## 1. Introduction

Raptors, as apex predators, are excellent bioindicators of their environment and the changes occurring in them whether by natural causes or owing to anthropogenic activities [1]. However, different factors can influence the sustainability of diurnal and nocturnal raptor populations, such as nest site availability [2], climate [3,4], and above all prey diversity and abundance [5]. Further, the fluctuation of prey-base can reflect changes, in the population dynamics of the prey species sensu stricto [6]. Thus, prey-base research can be used as tool in the monitoring of environmental change and the sustenance, not only of the apex predator populations, but also other groups of animals that are included in the prey-base. The diet of apex predators has been shown to affect reproduction in certain species (review in [7]; rock eagle owl, *Bubo bengalensis*, [8]; barn owl, *Tyto alba*, [9,10] while others found no relationships [11,12]). Thus, the effect of diet on breeding success remains unclear and is possibly not only species specific, but also may fluctuate within the species, between regions of its geographic distribution, and seasonally/annually [13,14,15]. Yet, to improve the conservation of a given species and its habitat, we have to improve our understanding of how diet influences recruitment and population sustainability [16]. This is especially important for diurnal and nocturnal raptors in as wide a range of study areas as possible, especially in regions such as the Middle East in general, and Israel in particular, where there is a lack of information of the prey-base, its effect on breeding success [13,14,15], and on the recruitment of young into the breeding population, and the sustenance of the existing population. Hence, we studied the diet and its possible influence on breeding success in a little studied species in Israel, the Eagle Owl (*B. bubo*, hereafter EO; [17]).

The diet of the EO can be extremely diverse, comprising many different species of mammals and birds, but also reptiles, amphibians, fish, carrion, and invertebrates [18,19,20,21,22]. As apex predators, EO are important for ecosystem functioning by direct or indirect effects such as by controlling other predator populations through depredation and the efficacy of trophic cascades [19,23,24]; or indirectly by other predators avoiding the territories of the EO [19]. In addition, EO are also important predators for a wide variety of prey, many of which, such as hedgehogs (*Erinaceinae* Sppl. [25,26,27,28,29,30,31]) or porcupines (*Hystrix indica*; [32]) are species which have a limited number of known predators.

The most common method of studying the diet of a wide range of species is by collecting and dissecting pellet contents. However, the subject of the reliability of pellet analyses is also questioned [33]. Although considered to be a valuable research technique to estimate prey diversity and as an index of specific species included in the diet, several drawbacks were noted. Mostly, that it is necessary to not only collect and analyze pellets, but also collect all prey remains in the vicinity of the nest. Pellets were found to over-represent smaller sized prey such as mammalian prey as compared to larger avian and mammalian prey. In comparison, prey remains over-represented large prey items. However, it was also found that neither did a combination of the two (pellets and prey remains) eliminate the biases [33,34]. Hence, biomass was considered to be a more representative parameter than the number of prey items included in the diet, and that displayed preferential feeding regimes in the study species.

EO breeding has been well studied in Europe with some studies concentrating on habitat characteristics [35,36,37], prey availability [38], and weather [39]. Even though the diet and breeding success of EO have been studied extensively throughout Europe [22], only diet has been studied in the Middle East [40,41]. Since the breeding [42] and diet [43] of Eagle Owls vary at different latitudes and longitudes regionally, so too do any relationships between them. Hence, we studied whether the diet of EO affects breeding success in central Israel. We believed the pairs in which mammalian prey predominated would have greater fledging success than those pairs that preyed mainly upon other prey species, including birds [10,18]. Moreover, we believed, due to the owl’s generalist hunting strategy, even though diet might be diverse and habitat-specific between the breeding pairs of EO, that we would be able to identify the major species included in their diet. Furthermore, since the study area is a gradient of habitats ranging from the Judean foothills to the Mediterranean coastline, we expected to find a geographic gradient of prey in the diet of the Eagle Owls [29].

## 2. Materials and Methods

### 2.1. Study Species

In Israel, the subspecies of EO is *B. b. interpositus,* which is known to breed in southern Europe and the Levant [17]. The species status at the national level is unclear and even though [17] EO is considered to be an uncommon resident breeder predominantly in the Mediterranean biomes in northern and central Israel, to date, no studies have been published on the species in Israel.

### 2.2. Study Area

**Figure 1:**
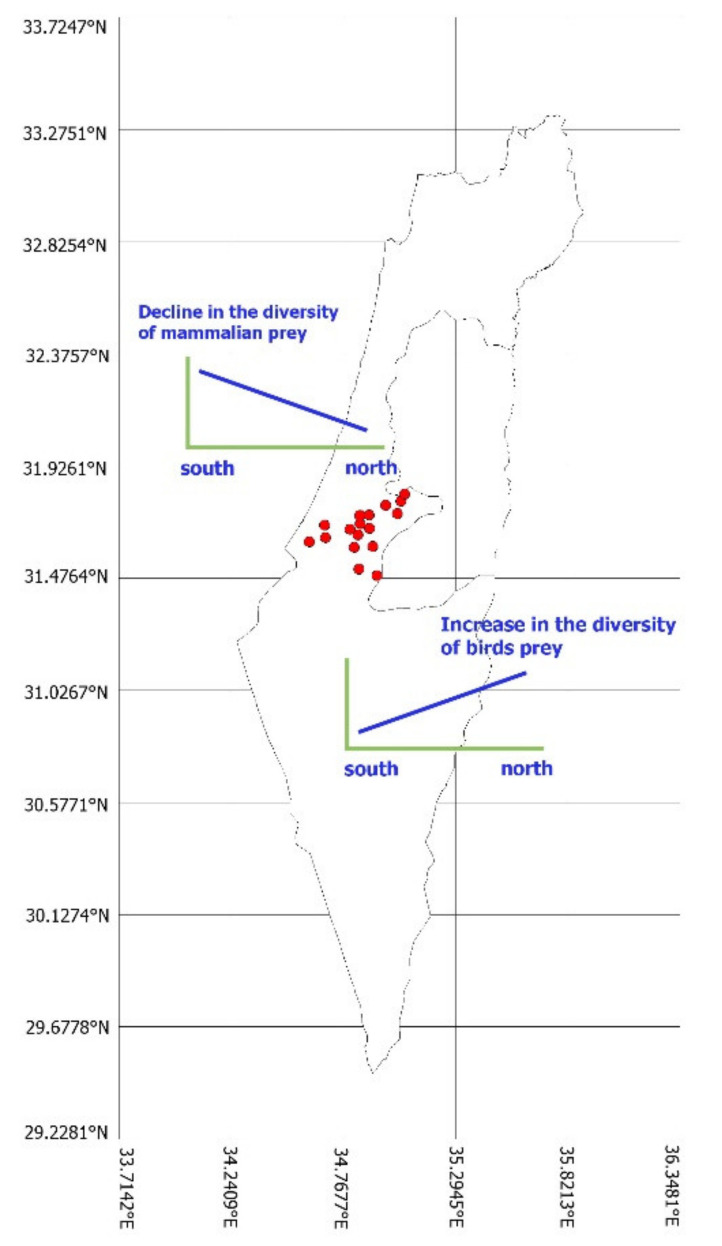
Map of Israel depicting the study area of Judea, central Israel, and the locations of the 14 nest sites (red circles) of Eagle Owls (*Bubo bubo*) at which pellets and prey remains were collected between 2006–2009.

The study was conducted in a 2644 km^2^ area of the Judea region of Israel (31°44′44.47″ N, 34°59′11.93″ E, Figure 1) during the four breeding seasons of 2006 to 2009. The average ±SE annual rainfall during 2006–2009 was 439 ± 44.3 mm (N = 4 years), with a mean ±SE daily maximum temperature of 27.2 ± 0.4 °C (N = 3) and mean ±SE daily minimum temperature of 12.5 ± 0.7 °C (N = 3 years) from 15 February to 15 July (data from Israel Meteorological Center). The region is mixed, with natural habitats (steppe and grasslands, N = 52.2% of the site), interspersed with agriculture (crop fields, vineyards, carob groves, N = 25.8%), human settlements (N = 10.6%), and planted pine tree forests (N = 11.4%).

### 2.3. Data Collection

The study site was visited weekly from March to July 2006 to 2009 by one of the authors (EH) and owl territories were identified by searching on foot in suitable habitats to detect EO activity (active nest sites, adults, feeding perches, fresh pellets; [44]), while also passively listening for calls [45,46] during the same period each year. We described an active nest site as one wherein the EO laid at least one egg; and a successful breeding attempt as when a pair fledged at least one young. During the study period, a total of 201 nest sites were found (Avg. 50.3 nests/season), and each nest was checked 5–8 times during the season to collect the pellets and prey remains.

Overall, 14 nests were monitored for pellets and prey remains: six of the nest sites were monitored in all four years, four nest sites for three years, one nest site for two years, and five nest sites for one year. We successfully collected pellets and prey remains from a cumulative of 43 nesting attempts in the four-year study period; 2006 (N = 10 nests), 2007 (N = 11 nests), 2008 (N = 11 nests), and 2009 (N = 11 nests). Both pellets and remains were collected so as to ensure that the sampling was not biased to small or large prey [34,47]. Prey biomass estimates were derived from data by [48,49], and avian prey from [50].

In order to calculate the relative percentage for each species, we calculated the average of the range of the body mass and multiplied by the number of individuals identified in the prey remains [33,34]. Prey specimens were identified to the species level [51].

### 2.4. Statistical Analysis

The biodiversity of prey at each nest was expressed as Shannon–Wiener diversity index:

$$H^{\prime }=-\overset{s}{\underset{i=1}{\sum }}\left(p_{i}\right)\left(log_{2}p_{1}\right)$$

where *H’* denotes the index of species diversity (calculated separately for the number and biomass), *s* the number of species, and *p_i_* the proportion of total samples belonging to *i*th.

To test for differences in H’ between particular groups (years, number of nestlings, etc.) we used one-way ANOVA. The normality of residuals coming from one-way ANOVA analysis was tested by the Shapiro–Wilk test, while Bartlett test was used as test of homogeneity variance. The Shapiro–Wilk test was also used to check that all dependent variables are in accordance with the normal distribution. If one of the above assumptions (i.e., normal distribution and homogeneity of variance) were not met, we used a non-parametric alternative to ANOVA, i.e., the Kruskal–Wallis test. All H’ indices showed accordance with the normal distribution (in all cases *p*-value for Shapiro–Wilk test was >0.05), while number of nestlings significantly deviates from the assumed theoretical normal distribution (*p* = 0.0023). In all cases, we used the Pearson coefficient for measurements of correlation.

## 3. Results

**Figure 2:**
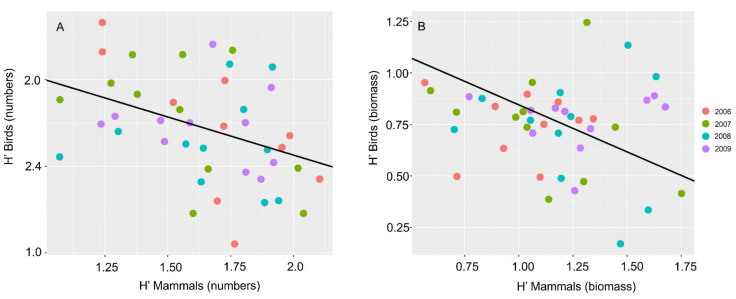
Relationship between the birds and mammals diversity in the diet of Eagle Owls during the 2006–2009 breeding seasons. (**A**): H’ for prey abundance, (**B**): biomass of birds and mammals.

The mean number of nestlings was similar between the 2006 (2.69 nestlings, range 2–4, N = 10), 2007 (2.71 nestlings, range 1–4, N = 11), 2008 (2.71 nestlings, range 1–4, N = 11), and 2009 breeding seasons (2.70 nestlings, range 1–4, N = 11). We calculated *H’* for all prey (h’all) found at nests as well as separately for avian (H’birds) and mammalian prey (H’mammals). All H’ indices (for numbers and biomass of prey) and the number of nestlings did not differ between years (one-way ANOVA, number of prey: H’all: F_3,39_ = 1.65, *p* = 0.19, H’mammals: F_3,39_ = 1.22, *p* = 0.31, H’birds: F_3,39_ = 0.34, *p* = 0.79, number of nestlings: Kruskal—Wallis test H_3_ = 1.42, *p* = 0.69). Furthermore, number of nestlings was also independent of nest geographical localization (Kruskal–Wallis test, longitude: H_3_ = 2.35, *p* = 0.502 and latitude: H_3_ = 2.87, *p* = 0.411). However, we found a relationship between the number of prey, i.e., mammals and birds (r = −0.40, *p* = 0.0060, Figure 2), and their biomass (r = −0.57, *p* < 0.001, Figure 2). Thus, to test our hypothesis, we used a linear mixed model [52] where nest id and year were used as random factors and all explanatory variables where normal distribution (in all cases, the *p*-value for Shapiro–Wilk test was >0.05). We used H’ (for all prey and mammals and birds separately in two variants, i.e., one for numbers of prey and the second for biomass) as the dependent variable, and longitude and latitude as explanatory variables. To test the significance of slope in the model, we used the *t*-test. We considered *p* < 0.05 as statistically significant. The statistical analyses were performed using R. 4.1.2.

Overall, 43 breeding attempts from 14 nest sites (mean 2.5 breeding attempts/nest + 0.39 SE) were monitored during the 2006–2009 breeding seasons (Table 1).

During the study, we identified 9461 prey specimens from the remains and pellets from 43 breeding attempts at the 14 nest sites. Numerically, mammals comprised the majority 72.89% (N = 6896, 35 species), followed by 23.83% birds (N = 2255, 55 species), 1.90% arachnids (N = 180, 9 species), 0.94% reptiles (N = 89, 6 species), 0.42% insects (N = 40, 5 species), and 0.01% amphibians (N = 1, 1 species; Table 2). However, when we calculated the average biomass of the vertebrate species based on the number of individuals, the ratios between the species changed (62.3% mammals, 36.0% birds, 1.7% reptiles, 0.1% amphibians).

Even though 111 prey species were identified, only the following seven mammal species made up more than 1% of the diet: Günther’s vole (*Microtus guentheri*; N = 2248, 23.8%, biomass 5.6%), Egyptian’s fruit bat (*Rousettus aegyptiacus*; N = 1103, 11.7%, biomass 2.0%), Tristram jird (*Meriones tristrami*; N = 998, 10.6%, biomass 3.9%), Southern white-breasted hedgehog (*Erinaceus concolor*; N = 720, 7.6%, biomass 4.2%), blind mole rat (*Nannospalax ehrenbergi*; N = 572, 6.0%, biomass 5.62%), black rat (*Rattus rattus*; N = 562, 5.9%, biomass 1.2%), brown rat (*Rattus norvegicus*; N = 295, 3.1%, biomass 1.4%); and four avian species, namely the Western jackdaw (*Coloeus monedula*; N = 538, 5.7%, biomass 6.1%), rock pigeon (*Columba livia*; N = 534, 5.6%, biomass 7.7%), chukar partridge (*Alectoris chukar*; N = 418, 4.4%, biomass 9.8%), and hooded crow (*Corvus cornix*; N = 140, 1.5%, biomass 3.9%). The only reptile species of significance was the javelin sand boa (*Eryx jaculus*; N = 77, 0.8%, biomass 1.4%); and of the Arthropoda was the large-clawed scorpion (*Scorpio maurus fuscus*; N = 144, 1.5%).

Predation on mesopredators (large carnivorous vertebrates) made up 3.0% of the diet including four species of Strigiformes (owls, 2.1% of the diet, N = 196), three species of Falconiformes (diurnal raptors, 0.7% of the diet, N = 65), and six species of Carnivora (0.25%, N = 24).

### 3.1. Diversity of Prey Numbers

The H’ for all prey, mammal, and birds did not have an effect on the number of nestlings (one-way ANOVA for numbers prey, respectively: H’all F_1,41_ = 1.52, *p* = 0.22; H’bird F_1,41_ = 0.34, *p* = 0.56; H’mammal F_1,41_ = 2.14, *p* = 0.15, Table 1).

**Figure 3:**
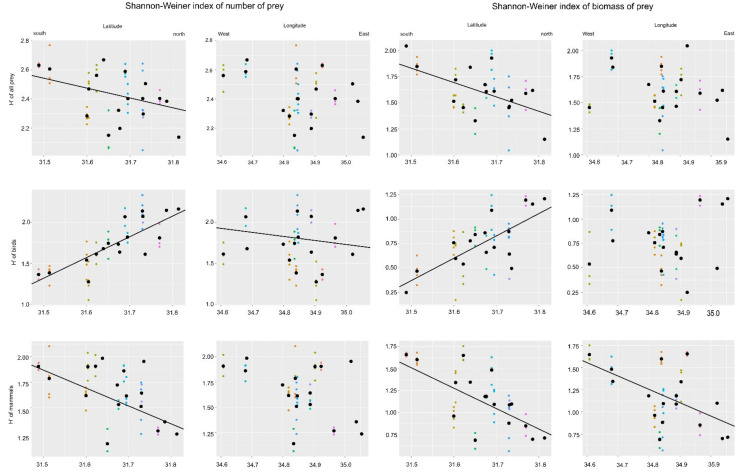
Relationship between longitude and latitude and the Shanon-Wiener Diversity Index for all prey, and birds and mammals. Circles of the same color denote the prey collected from the same nest of Eagle Owls (*Bubo bubo*) and black points indicate the mean value for the particular nest.

However, all H’ indexes changed linearly with longitude, while for the latitude we found no such relationship (Figure 3). Based on the linear mixed model for H’all, we discovered that H’all decreased from south to north (slope ± SE for this relationship was –0.68 ± 0.322, t = −2.11, *p* = 0.04), while longitude was not significant (−0.37 ± 0.254, t = −1.45, *p* = 0.153). The opposite relationship was found for H’birds. In this case, latitude was positively associated with this index (3.02 ± 0.36, t = 8.24, *p* < 0.001) but we also found decreased H’birds from east to west (−0.684 ± 0.290, t = −2.35, *p* = 0.023). For H’mammals, we found that this index decreased from south to north (−1.35 ± 0.44, t = −3.04, *p* = 0.004), while longitude was not significant (−0.699 ± 0.351, t = −1.99, *p* = 0.053).

### 3.2. Diversity of Prey Biomass

The biomass Shannon–Wiener diversity indices for all prey, mammal and birds had not effect on the number of nestlings (one-way ANOVA respectively: H’all F_1,41_ = 0.45, *p* = 0.50; H’bird F_1,41_ = 0.89, *p* = 0.34; H’mammal F_1,41_ = 4.07, *p* = 0.051. However, we found, similar to the H’ of numbers of prey, that biomass diversity index decreased from south to north (slope ± SE for this relationship was −1.32 ± 0.396, t = −3.34, *p* = 0.001), while longitude was not significant (0.059 ± 0.313, t = 0.19, *p* = 0.849). Avian biomass index showed an opposite relationship. In this case, this index increased from south to north (2.385 ± 0.414 t = 5.758, *p* < 0.001), while longitude was not significant (−0.236 ± 0.327, t = −0.723, *p* = 0.474). Mammalian biomass index also decreased from south to north (–−2.25 ± 0.477 t = −4.709, *p* < 0.001), and from west to east (−0.99 ± 0.77, t = −2.640, *p* = 0.011).

## 4. Discussion

Our results show that the prey base of the Eagle Owls breeding in central Israel shows different, compensatory gradients between the mammalian and avian prey-bases, wherein the biodiversity of the mammalian prey is the highest to the west and lowest to the east. Complementarily, the avian prey-base increases as mammalian prey decreases. This gradient is true for the biodiversity of mammalian prey, and in inverse relationship to the avian prey-base. The highest diversity of birds was to the east and the lowest in the west. Similarly, the mammal biomass diversity index decreased from south to north while the avian biomass index showed an increase from south to north. Ref [22] also reported such gradients for mammalian and avian prey, which was also affected by precipitation, altitude, variance among biomes, isothermality, and local conditions. A similar result was also found for Portugal wherein the prey-base composition varied along a gradient and no specific tendencies were obvious, and the authors thought this to be a consequence of the gradual landscape changes [29]. This may indeed also be the case in our study in which the landscape changes gradually from the Judean foothills to the Sharon Plain up to the Mediterranean coastline.

Ref [22] summarized breeding success for the species throughout its breeding distribution and found that the mean number of young fledged/successful pair ranged from 1 to 4, with fledging success ranging from 35% to 100%. This is similar to the breeding success found in Israel where the mean number of young fledged/successful pair ranged from 1–4, with fledging success ranging from 60–100%. However, it is of interest to note that, irrespective of the composition of the biodiversity of the prey-base, whether it is predominant mammal or avian, it does not influence the number of nestlings per breeding pairs. This suggests that the breeding pairs have adapted to this geographic trend of prey, i.e., there is a strong specialization for specific prey groups or species within each of the breeding pairs. This also suggests a relatively homogeneous population showing similar variance in productivity, which is similar to that described by [54].

Similar to other eagle owl studies [25,26,27,28,29,30,31], mammals (other studies = 74.6%, this study = 72.9%) and birds (other studies = 22.1%, this study = 23.8%) made up the majority of the prey. Undoubtedly, rodents make up an important part of the prey-base of the EO in the study area (N = 4814, biomass 17.6%). However, unlike other studies, rats did not make up a considerable portion of the diet in our study (2.6% for both species) as compared to as much as 80–90% in central France [55,56].

Avian prey is also an important prey-base for the more western pairs who appear to compensate for mammalian prey by taking a greater proportion of birds. This is especially true for prey species, such as chukars, pigeons, and corvids. Similar ratios were also reported for Portugal [29].

The study of the prey base in apex predators has been shown to demonstrate prey diversity and abundance, population levels and fluctuations, etc. [5,6]. Similarly, this study has highlighted several aspects, especially changes in the geographic distributions, of the prey species collected at the nests of the EO. For example, the debate of whether the Brown Rat was limited in Israel to the coastal regions only [57,58] was answered by the fact that they were also abundant in inland EO nests, indicating that the species is to be found also in the central Judea region. One of the eastern most of the nests monitored had a high concentration of 154 skulls found during two breeding seasons, and the answer probably lies in the fact that the nest is on the border of the Palestinian Authority and across the fence is located a regional garbage dump [59].

Another species that was a surprise to us because of its relatively high abundance at the EO nests was the Middle East blind mole-rat (*Nannospalax ehrenbergi;* N = 572; [53]). The majority were found at two nests showing a degree of specialization in these pairs on the hunting of this prey. The two pairs are at the southern extremes of our study site and in the vicinity has a high density of mole-rats. At one specific nest, we found 227 remains in the four seasons of our study, and at another 71 only in the 2008 season. The mole-rat lives in underground burrows and comes to the surface only at night, or when young during the dispersal period [60], which is when we assume they are preyed upon by the EO. Further, the finding of what are considered exclusively desert species, such as the Jerboa (*Jaculus jaculus*), was a surprise since they are a great distance away from their known distribution within the country. This is the first evidence for the species in the Judea region. Moreover, the marbled polecat (*Vormela peregusna*) is the smallest mammalian predator in Israel and is listed as Endangered in Israel and as Vulnerable in the International Union for the Conservation of Nature (IUCN) Red List [61]. The species was relatively widespread and common in Israel until the 1990s, when its numbers declined drastically and is now very rare [62,63]. The major threats to the species in Israel are secondary poising, loss of habitat, and road kills. However, finding six skulls in the prey remains of the EO is encouraging in that the species may be holding out in the open, agricultural mosaic landscape of the Judea region.

Bats typically make up a very small percentage of the diet of EO in Europe due to their small size and fast flight (>0.2%, [64]). They also found a statistically significant correlation between the weight of the owl species and the weight of the bats. They reported that EO take bast that average 21.5 g, while in our study the average body mass was 37.9 g (± 43.1 SD). Moreover, they found that in bats comprised as little as less than 0.2% of the diet while in our study it is 1.8%. This is probably because Egyptian fruit bats, which are medium-sized and common in Israel, were the second most numerous prey species in the owl’s diet (N = 1103) as compared to almost exclusively insectivorous species across the more northern latitudes in Eurasia. The exception was reported for bats caught on spring and autumn migration by EO in Far Eastern Russia, where they comprised as much as 50% of the diet during these periods [65].

Egyptian fruit bats do not have many predators in Israel and were in the past declared as agricultural pests, while extensive campaigns were conducted by the Ministry of Agriculture to eradicate the species from the 1950s till the late 1980s [66,67]. Roosts of the fruit bats are found in the vicinity of the EO nests, and their relatively large body size probably makes them an important prey species. Our study suggests that the EO supplies ecosystem services for the farmers in the region by diluting the fruit bat populations.

In addition to Egyptian fruit bats, the EO also preyed on nine different species, albeit in small numbers, especially of the insectivorous bats. The aforementioned campaign by the agricultural community on the fruit bats invariably also negatively affected all the other bat species. The Greater Horseshoe bat (*Rhinolophus ferrumequinum*; N = 38) is listed as Endangered in Israel [68] although a species of Least Concern with declining populations on the IUCN Red List [61], and our findings are a hopeful sign that the species may be recovering. In addition, finding remains of the Serotine bat (*Eptesicus serotinus*), a Mediterranean species listed as Endangered in Israel, is a sign that the species exists in the Judea region where it was considered to be extinct by the authorities. Two other bat species that appear to have enlarged their geographic distribution within Israel are the European free-tailed bat (*Tadarida teniotis*) and the Desert long-eared bat (*Otonycteris hemprichi*). Finding the Naked-rumped Tomb bat (*Taphozous nudiventris*) suggests its change in distribution from the Syrian-African Rift Valley towards the interior regions of Israel. Similarly, the Egyptian Tomb bat (*Taphozous perforatus*), a species of African origin and found only around the Dead Sea depression and considered extremely rare in Israel, appears to be extending its geographic range towards the Mediterranean. The same is also true for the Egyptian slit-faced bat (*Nycteris thebaica*) and the Greater mouse-tailed bat (*Rhinopoma microphyllum*), and this is the first evidence of these species from central Israel.

White-breasted hedgehogs made up 7.6% of the diet (biomass 4.2%), of the prey taken by EO in Israel. This is similar to Europe, where hedgehogs comprised on average 5.8% of the diet (range 0.1–13%) [25,26,27,28,29,30,31,59,69]. Since EO is the primary predator of White-breasted hedgehogs in Israel, they are most likely important in controlling their populations. However, it is of interest to note the finding of 14 skulls of long-eared hedgehogs (*Hemiechinus auritus*) because the known distribution of the species is further north and to the west of our study area, i.e., in the coastal regions from Caeserea to the sand dunes of the western Negev, and finding them in the prey items is evidence of their distribution also further inland in the Judea region. Similarly, the finding of Desert hedgehogs (*Paraechinus aethiopicus*) was also a surprise because their known distribution is much further to the south in the Negev Desert and to the East in the Judean Desert. Now, we have evidence that the species also occurs in the Judean region of Israel.

**Figure 4:**
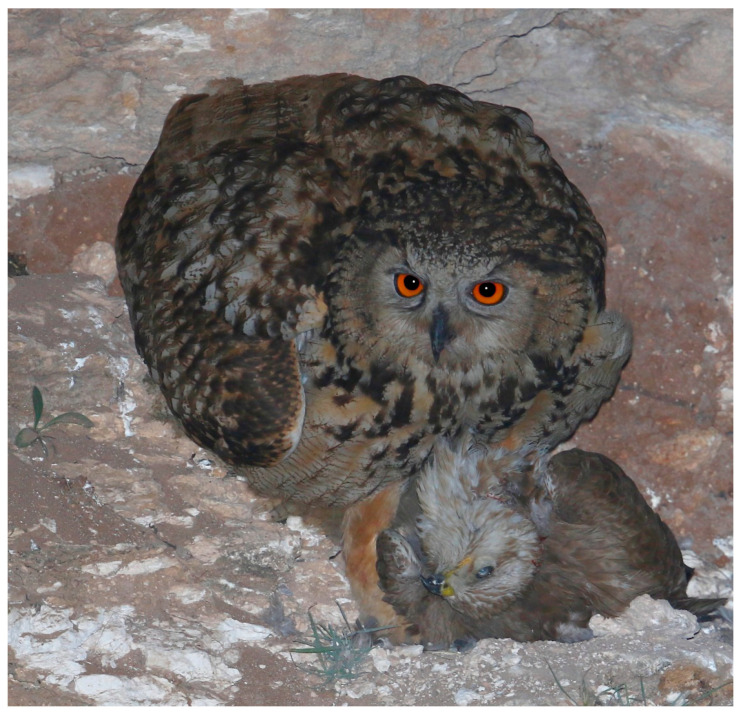
An adult Eagle Owl (*Bubo bubo*) brings a Steppe buzzard (*Buteo b. vulpinus*) to the nest. Photo: Ezra Hadad.

Even though EO are considered super-intraguild predators, and predated upon 18 different species of mesopredators, these species only made up a small percentage of the owls’ overall diet (N = 24, biomass 0.42%), which is in agreement with other studies [18,19,20,21,22]. Hence, although mesopredators are obviously preyed upon opportunistically, they are probably not an important energetic resource in the breeding effort. However, the presence of mesopredators is most likely related to a decrease in other prey species and to the proximity of breeding mesopredators nests to EO [18,21]. Although predation by Eagle Owls on mesopredators might make up a small part of the eagle owls’ diet (Owls N = 172, biomass 2.3%; raptors N = 89, biomass 2.3%), this predation might still be important in controlling competing populations through both direct and indirect effects [17,23] (Figure 4).

Especially of interest is the predation by EO on the larger raptors, namely the Long-legged buzzard (LLB) and Short-toed Eagle (StE). We have observed not only nestlings (seven LLB, five StE) being taken from their nests in both the raptor species, but three cases (two LLB, one StE) where the EO also killed and brought the carcass of the brooding female to feed its nestlings at the nest site. Following the installation of closed-circuit television (CCTV) cameras in recent years, this phenomenon has been substantiated in real time whereby EO have been documented nest-robbing nestlings as well as hunting adults in the nest site.

**Figure 5:**
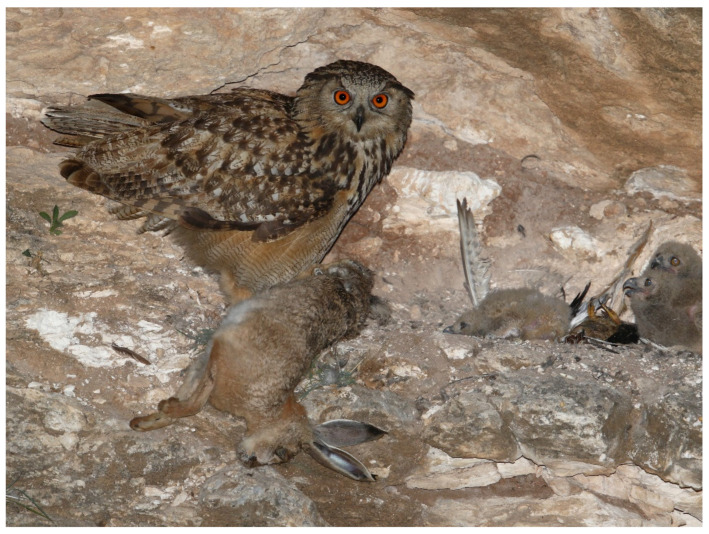
Adult Eagle Owl (*Bubo bubo*) brings a Cape Hare (*Lepus capensis*) to its’ nestlings. Note remains of a Common Kestrel (*Falco tinnunculus*) in the background. Photo: Ezra Hadad.

Unlike some of the European studies, from comparable biomes, wherein Lagomorphs comprised a major part of the diet (27–89%) and sustained EO populations [29,70,71,72,73], this was not the case in our study. Cape Hares, although relatively numerous (N = 87, biomass 1.8%; Figure 5), were not the main prey at any of the breeding sites in our study. Similarly, owing to the fact that our study is a Mediterranean shrub-habitat with a relatively stable climate, we do not think that there are marked seasonal or between year changes in the prey-base, as evidenced in northern latitudes where Lagomorph and vole population cycles dictate breeding success [29].

There is a need for further studies to determine whether the relatively high breeding success in central Israel is related to prey abundance and/or the owls’ ability to be opportunistic hunters. Future studies should try to understand at the micro- and macro-scales the influences of habitat characteristics on the prey-base available to the EO. Further, in light of the relatively high number of diurnal and nocturnal raptors included in their prey-base, it will be of interest to understand the influence EO have on the other raptorial species in the vicinity of their breeding site or hunting territory.

Further, although we have attempted to collect all pellets and prey remains at each of the visits to the nests [28], we did not use multiple techniques (e.g., visual, camera-traps, etc.; [33]) to resolve whether all of the prey taken are also represented in the samples collected. We assume that, based on the optimal-foraging theory, the soft-bodied prey taken by the adults are either consumed by them owing to their small size; and we assume that the majority of the pellets found at the nest are those of the nestlings, which are mostly fed the larger prey, and which is cost-effective for the parent to handle and transport to the nest [74,75,76]. Hence, future studies should also try to incorporate different techniques to try and elucidate the ratio of prey taken that are not represented in the pellets of the EO.

In conclusion, our study, based on a large sample size of prey remains and pellets, demonstrates the generalist hunting behavior of the Eagle Owls and the opportunistic manner in which they appear to take their prey. We have also shown that there is a latitudinal gradient in the prey-base with mammals being predominant to the west, which is complimentarily compensated by an increasing proportion of avian prey. We assume that this generalist and flexible manner of hunting prey is a strategy that allows this apex predator to subsist in the human-congested and well-developed region of central Israel.

## Figures and Tables

**Table 1:** Results of the Shanon-Wiener Diversity Index for the effect of prey-base composition on prey diversity and breeding success in Eagle Owl (*Bubo bubo*) in the Judea region, central Israel.

Year	Number of Nestlings (±SE)	H’all (±SE)	H’mammals (±SE)	H’birds (±SE)	Biomass H’all (±SE)	Biomass H’mammals (±SE)	Biomass H’birds (±SE)
2006	2.68 ± 0.28	2.39 ± 0.05	1.63 ± 0.08	1.76 ± 0.09	1.59 ± 0.06	1.14 ± 0.10	0.73 ± 0.08
2007	2.71 ± 0.26	2.40 ± 0.08	1.63 ± 0.08	1.73 ± 0.09	1.59 ± 0.06	1.15 ± 0.09	0.74 ± 0.08
2008	2.72 ± 0.27	2.42 ± 0.05	1.66 ± 0.08	1.69 ± 0.09	1.63 ± 0.06	1.17 ± 0.10	0.75 ± 0.08
2009	2.71 ± 0.26	2.41 ± 0.05	1.67 ± 0.08	1.68 ± 0.09	1.62 ± 0.06	1.16 ± 0.10	0.75 ± 0.08
all	2.67 ± 0.13	2.41 ± 0.02	1.64 ± 0.04	1.71 ± 0.04	1.61 ± 0.03	1.17 ± 0.05	0.78 ± 0.04

**Table 2:** The diet determined according to pellet remains collected from 14 nests, i.e., 43 breeding attempts, of Eagle Owls during the 2006–2009 breeding seasons in Israel. Total biomass was calculated by the average body mass of the species from the literature multiplied by the number of items. Mammal taxonomy based on [52,53].

			Prey Specimens	Number	% of Total Prey	Body Mass (g, Range)	Total Biomass (g)	% Biomass
Vertebrata							(Average)	
	Mammals	Insectivora						
		Erinaceidae	*Erinaceus concolor*	720	7.61%	350–880 (492.0)	354,240.0	4.2
			*Hemiechinus auritus*	14	0.15%	131–358 (244.5)	3423.0	0.04
			*Paraechinus aethiopicus*	1	0.01%	227–605 (416.0)	416.0	0.004
		Soricidae	*Crocidura* spp.	1	0.01%	1.8–3.6 (2.7)	2.7	0.001
			*Crocidura suaveolens*	60	0.63%	2.4–8.5 (5.5)	330.0	0.01
			*Crocidura leucodon*	14	0.15%	7–15 (10.5)	147.0	0.008
		Chiroptera						
		Pteropodidae	*Rousettus aegyptiacus*	1103	11.66%	90–210 (150.0)	165,450.0	2
		Rhinopomatidae	*Rhinopoma microphyllum*	2	0.02%	16–24 (20.0)	40.0	0.002
		Emballonuridae	*Taphozous nudiventris*	3	0.03%	30–60 (45.0)	135.0	0.007
			*Taphozous perforatus*	2	0.02%	22–33 (27.5)	55.0	0.002
		Nycteridae	*Nycteris thebaica*	1	0.01%	8–14 (11.0)	11.0	0.0005
		Rhinolophidae	*Rhinolophus ferrumequinum*	35	0.37%	10–20 (15.0)	525.0	0.03
		Vespertilionidae	*Eptesicus serotinus*	1	0.01%	20–33 (26.5)	26.5	0.01
		Hipposideridae	*Otonycteris hemprichii*	5	0.05%	16.5–23 (20.0)	100.0	0.3
		Molossidae	*Tadarida teniotis*	9	0.10%	14–38 (26.0)	234.0	0.01
		Lagomorpha						
		Leporidae	*Lepus capensis*	87	0.92%	1250–2300 (1775.0)	154,425.0	1.8
		Rodentia						
		Cricetidae	*Cricetulus migratorius*	1	0.01%	18–35 (26.5)	26.5	0.001
			*Microtus guentheri*	2248	23.76%	29–69 (49.0)	110,152.0	5.6
		Muridae	*Meriones tristrami*	998	10.55%	48–104 (76.0)	75,848.0	3.9
			*Apodemus mystacinus*	26	0.27%	20–57 (38.5)	1001.0	0.05
			*Rattus rattus*	562	5.94%	100–200 (150.0)	84,300.0	1.2
			*Rattus norvegicus*	295	3.12%	200–600 (400.0)	118,000.0	1.4
			*Mus musculus*	58	0.61%	8–15 (11.5)	667.0	0.03
			*Gerbillus dasyurus*	18	0.19%	15–34 (24.5)	441.0	0.02
			*Acomys dimidiatus*	30	0.32%	26–57 (41.5)	1245.0	0.06
		Spalacidae	*Nannospalax ehrenbergi*	572	6.04%	118–240 (179.0)	102,388.0	5.2
		Dipodidae	*Jaculus jaculus*	1	0.01%	33–91 (63.5)	63.5	0.003
		Hystricidae	*Hystrix indica*	2	0.02%	2000	4000.0	0.05
		Echimyidae	*Myocastor coypus*	3	0.03%	1000–2000 (1500.0)	4500.0	0.05
		Carnivora						
		Mustelidae	*Meles meles*	1	0.01%	2000–3000 (2500.0)	2500.0	0.03
			*Vormela peregusna*	6	0.06%	200–500 (350.0)	2100.0	0.03
			*Martes foina*	2	0.02%	700–1300 (1000.0)	2000.0	0.03
		Canidae	*Vulpes vulpes*	7	0.07%	2200	15,400.0	0.2
		Herpestidae	*Herpestes ichneumon*	1	0.01%	2000	2000.0	0.03
		Felidae	*Felis catus*	7	0.07%	1500	10,500.0	0.1
	Birds							
		Pelecaniformes						
		Ardeidae	*Nycticorax nycticorax*	1	0.01%	500–800 (650.0)	650.0	0.03
			*Bubulcus ibis*	3	0.03%	300–400 (350.0)	1050.0	0.05
		Threskiornithidae	*Plegadis falcinellus*	1	0.01%	530–760 (645.0)	645.0	0.03
		Anaseriformes						
		Anatidae	*Anas platyrhynchos*	6	0.06%	750–1450 (1100.0)	6600.0	0.3
			*Spatula clypeata*	1	0.01%	470–750 (310.0)	610.0	0.03
			*Anas crecca\Spatula querquedula*	7	0.07%	200–450 (325.0)	2275.0	0.1
			*Anatidae* sp.	2	0.02%	200–450 (325.0)	650.0	0.03
		Accipitriformes						
		Accipitridae	*Circaetus gallicus*	6	0.06%	1200–2200 (1700.0)	10,200.0	0.5
			*Circus pygargus*	1	0.01%	227–445 (336.0)	336.0	0.02
			*Buteo rufinus*	9	0.10%	590–1760 (1175.0)	10,575.0	0.5
			*Buteo (buteo) vulpinus*	10	0.11%	550–1300 (925.0)	9250.0	0.5
			*Accipiter nisus*	5	0.05%	110–342 (226.0)	1130.0	0.1
		Falconiformes						
		Falconidae	*Falco tinnunculus*	55	0.58%	156–252 (204.0)	11,220.0	0.6
			*Falco naumanni*	1	0.01%	90–208 (149.0)	149.0	0.002
			*Falco subbuteo*	2	0.02%	131–340 (235.5)	471.0	0.03
		Galliformes						
		Phasianidae	*Alectoris chukar*	418	4.42%	360–560 (460.0)	192,280.0	9.8
			*Coturnix coturnix*	9	0.10%	75–135 (105.0)	945.0	0.05
			*Gallus gallus domesticus*	3	0.03%	1000	3000.0	0.2
		Gruiiformes						
		Rallidae	*Rallus aquaticus*	1	0.01%	80–180 (130.0)	130.0	0.002
			*Porzana porzana*	1	0.01%	70–110 (90.0)	90.0	0.004
			*Gallinula chloropus*	6	0.06%	240–420 (330.0)	1980.0	0.1
			*Fulica atra*	7	0.07%	700–1000 (850.0)	5950.0	0.3
			*Crex crex*	6	0.06%	120–200 (160.0)	960.0	0.05
		Charadriiformes						
		Recurvirostridae	*Himantopus himantopus*	2	0.02%	150–210 (180.0)	360.0	0.02
		Burhinidae	*Burhinus oedicnemus*	48	0.51%	430–500 (465.0)	22,320.0	1.1
		Charadriidae	*Vanellus spinosus*	37	0.39%	127–159 (145.0)	5365.0	0.3
		Scolopacidae	*Tringa* spp.	4	0.04%	40–70 (55.0)	220.0	0.01
			*Numenius arquata*	1	0.01%	540–1300 (920.0)	920.0	0.05
		Glareolidae	*Cursorius cursor*	3	0.03%	102–119 (110.5)	331.5	0.02
		Laridae	*Larus* spp.	1	0.01%	600–940 (770.0)	770.0	0.04
		Pterocliformes						
		Pteroclidae	*Pterocles orientalis*	1	0.01%	300–550 (425.0)	425.0	0.03
		Columbiformes						
		Columbidae	*Columba livia*	534	5.64%	230–370 (280.0)	149,520.0	7.7
			*Streptopelia* spp.	54	0.57%	80–240 (160.0)	8640.0	0.4
		Cuculiformes						
		Cuculidae	*Clamator glandarius*	3	0.03%	138–192 (165.0)	495.0	0.03
		Strigiformes						
		Tytonidae	*Tyto alba*	71	0.75%	240–350 (295.0)	20,945.0	1.1
		Strigidae	*Asio otus*	66	0.70%	220–370 (295.0)	19,470.0.0	1
			*Athene noctua*	16	0.17%	140–220 (180.0)	2880.0	0.1
			*Otus scops*	19	0.20%	60–120 (90.0)	1710.0	0.1
		Caprimulgiformes						
		Caprimulgidae	*Caprimulgus europaeus*	2	0.02%	65–100 (82.5)	165.0	0.002
		Bucerotiformes						
		Upupidae	*Upupa epops*	2	0.02%	47–87 (67.0)	134.0	0.002
		Coraciiformes						
		Alcedinidae	*Halcyon smyrnensis*	3	0.03%	85–110 (97.5)	292.5	0.003
		Meropidae	*Merops apiaster*	11	0.12%	44–78 (61.0)	671.0	0.03
		Coraciidae	*Coracias garrulus*	24	0.25%	120–160 (140.0)	3360.0	0.2
		Psittaciformes						
		Psittaculidae	*Psittacula krameri*	2	0.02%	96–139 (120.0)	240.0	0.003
		Passeriformes	*Passeriformes* spp.	62	0.66%	10–25 (17.5)	1085.0	0.01
		Alaudidae	*Galerida cristata*	1	0.01%	37–55 (46.0)	46.0	0.005
		Motacillidae	*Motacilla alba*	1	0.01%	17–25 (21.0)	21.0	0.003
		Turdidae	*Turdus merula*	1	0.01%	80–125 (102.5)	102.5	0.001
		Laniidae	*Lanius senator*	2	0.02%	30–40 (35.0)	70.0	0.008
			*Lanius nubicus*	1	0.01%	22–30 (26.0)	26.0	0.0003
			*Lanius excubitor*	2	0.02%	48–81 (64.5)	129.0	0.002
		Corvidae	*Garrulus glandarius*	41	0.43%	140–190 (165.0)	6765.0	0.08
			*Coloeus monedula*	538	5.69%	180–260 (220.0)	118,360.0	6.1
			*Corvus cornix*	140	1.48%	430–650 (540.0)	75,600.0	3.9
		Oriolidae	*Oriolus oriolus*	1	0.01%	56–79 (67.5)	67.5	0.008
	Reptiles	Squamata						
		Boidae	*Eryx jaculus*	77	0.81%	350	26,950.0	1.4
		Viperidae	*Daboia palaestinae*	4	0.04%	1500	6000.0	0.3
		Colubridae	*Rhynchocalamus melanocephalus*	1	0.01%	20	20.0	0.002
			*Hemorrhois nummifer*	1	0.01%	600	600.0	0.003
		Lamprophiidae	*Micrelaps muelleri*	2	0.04%	25	50.0	0.003
		Testudines						
		Testudinidae	*Testudo graeca*	2	0.02%	60	120.0	0.002
	Amphibian	Anura						
		Bufonidae	*Bufotes variabilis*	1	0.01%	25	25.0	0.002
Invertebrata								
	Insecta		*Insecta* spp.	2	0.02%			
		Gryllotalpidae	*Gryllotalpa gryllotalpa*	21	0.22%			
		Cerambycidae	*Cerambyx dux*	6	0.06%			
			*Orthoptera* spp.	6	0.06%			
			*Coleoptera* spp.	5	0.05%			
	Arachnida	Scorpiones	*Scorpiones* sp.	1	0.01%			
		Buthidae	*Leiurus hebraeus*	2	0.02%			
			*Buthotus judaicus*	1	0.01%			
			*Androctonus bicolor*	2	0.02%			
		Scorpionidae	*Scorpio palmatus*	3	0.03%			
			*Scorpio fuscus*	144	1.52%			
		Diplocentridae	*Nebo hierichonticus*	10	0.11%			
		Solifugae	*Solifugae* spp.	17	0.18%			
	Crustacea	Potamidae	*Potamon potamios*	2	0.02%			
Total Prey				9461				

## Data Availability

Data is contained within the article.
